# Worksite Health Promotion: Principles, Resources, and Challenges

**Published:** 2009-12-15

**Authors:** Phillip B. Sparling

**Affiliations:** School of Applied Physiology, Georgia Institute of Technology

Employee health has long been a priority of owners of businesses, small and large. As health care costs continue to escalate, the demand for worksite health promotion programs that improve workers' health and provide a return on investment has never been greater. However, as we close the first decade of the 21st century, the future of worksite health promotion — also known as *worksite wellness* and more recently *employee health and productivity programs* — remains uncertain.

The purpose of this essay is not to provide a research review or status report on worksite health promotion, as these are available elsewhere (1,2). Rather, the purpose is to remind us that successful worksite health promotion depends on the cooperation and collaboration of many different stakeholders and sectors. The importance of divergent groups working together is illustrated by examining the recent history of worksite health promotion and highlighting key underlying principles and information resources.

## A Brief Historical Overview

Many of today's worksite health promotion programs originated from executive fitness programs that were created in the years after World War II. Initiated by business leaders who endorsed the benefits of a healthful lifestyle, the number of in-house corporate programs grew steadily throughout the 1970s. Well-appointed gyms staffed with fitness instructors and masseurs were standard fare for successful companies. These perks were typically restricted to upper management and, therefore, had little influence on health behaviors or health care provisions of most employees. However, from these beginnings, the seeds of worksite health promotion were sown (3,4).

In the mid-1970s, the American Association of Fitness Directors in Business and Industry — known later as the Association for Fitness in Business — was organized to provide networking and conferences (N. Pronk, HealthPartners, personal communication, October 2008). During the next decade, employer benefits began to focus on broad health issues instead of focusing on fitness and were increasingly offered to employees of all job levels. Companies changed organizationally; they established new links between occupational medicine and human resources and strengthened previous relationships. The Association for Fitness in Business, a proponent of the shift from corporate fitness to worksite wellness, became the Association for Worksite Health Promotion.

By the early 1990s, the Association for Worksite Health Promotion had more than 2,500 members and operated at national and regional levels. Then, because of widespread economic pressures and the resulting corporate restructuring, the association ceased to exist as a stand-alone organization. The adoption of the worksite health promotion agenda by the American College of Sports Medicine and its Interest Group on Worksite Health Promotion allowed for continued scaled-down service. In 2009, this interest group expanded into a new affiliate association, the International Association for Worksite Health Promotion (www.iawhp.org). Hopefully, this expansion marks a resurgence in workplace health programs.

From the early days of executive fitness programs to today's multifaceted wellness programs, support of worksite health has understandably been tied to the fiscal health of the company. However, employee health is now recognized as more than an expendable fringe benefit. Workforce health is an essential element in determining the long-term success of a company and whether it thrives. Companies must focus on the prevention, screening, and management of prevalent chronic conditions including obesity, diabetes, heart disease, cancer, and depression. Part of the solution is to implement programs that improve multiple behavioral risk factors (eg, smoking, unhealthy diet, sedentary lifestyle) of employees and their families (5-7).

## Multiple Approaches to a Common Challenge

Federal agencies and private sector groups — nonprofit and for-profit — are working hard to help employers improve the health of their employees in an efficient, integrated, and cost-effective way. The objective is clear; it is the "how to" that is difficult. However, some groups, agencies, and coalitions have developed and are disseminating practical solutions.

The Centers for Disease Control and Prevention (CDC) has been a leader during the past decade in identifying and translating science-based strategies to help employers improve the health of their employees. The emphasis has been on strategies that are effective, feasible, and cost-effective in worksite settings. In collaboration with experts in research, practice, and policy, CDC staff conduct systematic reviews of published scientific studies on various topics, including worksite health promotion, for the Task Force on Community Preventive Services. The Task Force, a nonfederal volunteer group of public health and prevention experts, bases its findings and recommendations for evidence-based public health interventions on these systematic reviews. Their recommendations are available in the *Guide to Community Preventive Services* (*Community Guide*) (8).

In a parallel effort, CDC provides the Healthier Worksite Initiative Web site (9) as a resource for worksite health promotion program planners in state and federal government, although many recommended strategies can also be applied in nongovernment workplaces. Moreover, the National Institute for Occupational Safety and Health (NIOSH, an agency in CDC) recently released the *Essential Elements of Effective Workplace Programs and Policies for Improving Worker Health and Wellbeing* (10), a document that identifies components of a comprehensive work-based program that incorporates both health promotion and health protection.

CDC also collaborated with the National Business Group on Health in developing *A Purchaser's Guide to Clinical Preventive Services* (11). This in-depth document translates clinical recommendations for employers, so they can make informed decisions when selecting medical services (eg, cancer screening, smoking cessation intervention) offered by health insurance carriers.

Established in the mid-1980s, the Wellness Council of America (WELCOA) is a nonprofit organization that supports health promotion initiatives at the worksite. Oriented toward the business community, WELCOA shares materials, resources, and networking opportunities. WELCOA also recognizes outstanding worksite health promotion programs annually through Well Workplace Awards (12). A similar recognition program is the C. Everett Koop National Health Award (13). In both award programs, winners are profiled, and innovations and successes are shared.

In academia, scholars continue to test conceptual models to better understand the dynamics of health management (14). The fundamental proposition that guides investigators is that recommendations be grounded in good science. However, the meaning of "good science" is evolving, particularly when the aim is to modify human behavior. We can no longer depend mainly on expensive and multiyear research designs such as randomized clinical trials; the cost in dollars and time is too great. To address complex questions about employee health, we should be open to all types of evidence and the merits of divergent evaluative approaches, as long as rigor and standards are maintained (15,16).

An example of a different approach is the Swift Worksite Assessment and Translation (SWAT) project (17), which used a field-based approach to learn directly from the business community. Its basis was the supposition that some employers — particularly small to medium-sized organizations — develop successful health promotion practices through their own innovation, ingenuity, and perseverance. The SWAT approach — a rapid assessment method — was designed to uncover field-based practices that were promising and explored practice-based evidence to complement evidence-based recommendations from the *Community Guide*.

Over the years, coalitions have advanced our understanding of worksite health. The National Business Group on Health and the Partnership for Prevention, both based in Washington, DC, are 2 prominent examples. Health scientists, business leaders, and health insurers have different perspectives and different objectives. Coalitions of stakeholders provide the opportunity to identify common goals using a common language and to develop an action plan that benefits everyone. The need for the public health community to create strong and lasting partnerships with the business sector has never been greater (18).

## Key Principles to Consider

Successful workplace initiatives often differ in their approaches to improve employee health and contain health care costs, but many time-tested and accepted principles consistently emerge (19,20). Although the relevance or application of these principles varies among worksites, all principles are useful for business leaders and worksite health professionals to consider when reviewing an organization's health promotion program, including its philosophy, scope, and services (3,4,21).

**Principle 1: Successful worksite health promotion programs have multiple components and strive to be comprehensive and integrated.** According to *Healthy People 2010* (22), 5 elements of a comprehensive worksite health program are health education, supportive social and physical environments, integration of the worksite program, linkage to related programs, and screening programs. Successful worksite health promotion programs recognize the interrelatedness of disease prevention and disease management and strive to encompass both in a unified approach. Employee health insurance coverage, worker safety programs, and occupational medicine should be integrated with worksite health promotion.

**Principle 2: Successful worksite health promotion programs demonstrate visible and unequivocal commitment to employee health through the actions of the top leadership of the organization.** This commitment of leadership is clearly stated and included as a guiding principle of the organization. Typically (but not always), this steadfast commitment to a healthy workforce is the result of a business decision made based on the premise that investing in employee health will reduce health care costs. Other benefits, such as decreased absenteeism, increased productivity, and high employee satisfaction, are also cited as reasons by business leaders. True concern and action by employers for the welfare of employees can be a powerful influence on employee morale, loyalty, and retention.

**Principle 3: Successful worksite health promotion programs are open to all employees.** Programs that cater only to top management are no longer acceptable. Programs should be open to all workers from all job categories and locations. If health care costs — and worksite culture — are to be meaningfully affected, wellness programs must be designed to reach all. Challenges include how to provide services to employees located at different sites or who are located off-site and to retirees.

**Principle 4: Successful worksite health promotion programs provide systematic health assessments, timely and meaningful feedback, and assistance in setting and monitoring individual health goals.** This principle is central to worksite health promotion. Meaningful feedback and regular follow-up are key to helping employees make and sustain healthy behaviors. This process is most effective when rapport is established between professional staff and employees, and a supportive social network emerges among fellow employees.

**Principle 5: Successful worksite health promotion programs tailor health promotion activities to the needs of the employees.** Employee needs vary by age, sex, education, type of industry, job category, ethnicity and cultural factors, and geographic location. Identifying health needs of employees should be done systematically and should include input from the employees. Tailoring health promotion refers to both providing needed services and delivering them in a relevant, engaging way.

**Principle 6: Successful worksite health promotion programs attain high participation by using creative and appealing incentive-based programs.** Because traditional elective programs have been unable to achieve high levels of employee participation, incentive-based and opt-out (instead of opt-in) programs are being tested and adapted to ensure some level of participation by most employees. For example, many companies "incentivize" health promotion services by offering lower health insurance premiums. The aim is to have all employees see health promotion services as an attractive company benefit.

**Principle 7: Successful worksite health promotion programs implement and sustain environmental and policy changes that support healthy behaviors.** Examples include attractive stairwells with prompts; worksite food services that offer healthful choices; provision of shower and change areas for employees who wish to walk, jog, or cycle to work; and a policy of offering healthful foods at meetings. Multiple policies can have a cumulative effect over time that moves the worksite culture toward a place in which healthy behaviors are the norm, not the exception.

**Principle 8: Successful worksite health promotion programs link health promotion services to occupational safety and job performance at all employee levels.** Showing the relevance of good health to worksite safety practices and having supervisors reinforce this concept with their employees are critical. Workers who follow safety practices are less prone to error and injuries and are more productive. If those employees are healthy (eg, alert, energetic) to begin with, they likely will work more safely and efficiently. These programs — health insurance/medical benefits, worksite safety, and health promotion — are synergistic; all contribute to employee health.

**Principle 9: Successful worksite health promotion programs actively extend health promotion services to spouses and family members.** Health care costs are routinely determined by the health needs of the employee and those of his or her family. Moreover, health behaviors are shaped by families and social networks. Therefore, companies are wise to extend services to families. An additional step is for companies to support health projects in local communities to demonstrate their corporate commitment to healthy living.

**Principle 10: Successful worksite health promotion programs systematically evaluate employees' health needs and the effectiveness of health promotion services and activities in meeting these needs.** Most decisions in business (as in science) are data-driven. The top programs regularly evaluate how well they are doing and use their findings to adapt and improve. Lack of systematic and regular evaluation is the Achilles' heel of many programs. Evaluation is a critical piece of health promotion and should be incorporated into the overall design of a company's program.

## Moving Forward Together to Improve Workers' Health

**Box. Noncommercial Web Sites on Worksite Health Promotion**
CDC Healthier Worksite Initiative: www.cdc.gov/nccdphp/dnpa/hwi
NIOSH WorkLife Initiative: www.cdc.gov/niosh/worklife/essentials.html
The *Community Guide*: www.thecommunityguide.org/worksite
National Business Group on Health: www.businessgrouphealth.org
Partnership for Prevention: www.prevent.org
Wellness Council of America: www.welcoa.org
The Health Communication Unit (Canada): www.thcu.ca

These 10 principles illustrate fundamental characteristics of successful worksite health promotion programs, and all are supported by some level of evidence. This list is by no means exhaustive; rather, it is a condensed version of core principles. Much of the work to be done involves implementing principles such as these, while advancing the research agenda to expand the science base and recommendations. By regularly visiting trusted, noncommercial Web sites (Box), readers can review guidelines, identify useful strategies, and stay informed.

Our overarching aim is to improve the health and productivity of America's workforce. This daunting challenge requires multi-sector collaborations that can only succeed through the development of trust between all parties and the sustained commitments of resources to shared goals. Progress is being made. Effective partnerships are growing and strengthening. Let us all — health care practitioners, health education specialists, health policy experts, business owners, health insurers, and employees — reaffirm our resolve to work together and be steadfast in our collective enterprise. At the grass-roots level, the promise of worksite health promotion involves sharing experiences and applying key principles now.
